# Effect of librarian collaboration on otolaryngology systematic review and meta-analysis quality

**DOI:** 10.5195/jmla.2024.1774

**Published:** 2024-07-01

**Authors:** Rachel Whitney, Michael C. Shih, Tamar Gordis, Shaun A. Nguyen, Ted A. Meyer, Emily A. Brennan

**Affiliations:** 1 Research Informationist, Medical University of South Carolina Libraries, Charleston, SC, 29425.; 2 Department of Internal Medicine, Tulane University, New Orleans, LA, 70112.; 3 Department of Otolaryngology – Head & Neck Surgery, Medical University of South Carolina, Charleston, SC, 29425.; 4 Evidence Synthesis Informationist, Medical University of South Carolina Libraries, Charleston, SC, 29425.

**Keywords:** Systematic reviews, meta-analyses, otolaryngology, librarians, reproducibility

## Abstract

**Objective:**

To determine if librarian collaboration was associated with improved database search quality, search reproducibility, and systematic review reporting in otolaryngology systematic reviews and meta-analyses.

**Methods:**

In this retrospective cross-sectional study, PubMed was queried for systematic reviews and meta-analyses published in otolaryngology journals in 2010, 2015, and 2021. Two researchers independently extracted data. Two librarians independently rated search strategy reproducibility and quality for each article. The main outcomes include association of librarian involvement with study reporting quality, search quality, and publication metrics in otolaryngology systematic reviews and meta-analyses. Categorical data were compared with Chi-Squared tests or Fisher's Exact tests. Continuous variables were compared via Mann Whitney U Tests for two groups, and Kruskal-Wallis Tests for three or more groups.

**Results:**

Of 559 articles retrieved, 505 were analyzed. More studies indicated librarian involvement in 2021 (n=72, 20.7%) compared to 2015 (n=14, 10.4%) and 2010 (n=2, 9.0%) (p=0.04). 2021 studies showed improvements in properly using a reporting tool (p<0.001), number of databases queried (p<0.001), describing date of database searches (p<0.001), and including a flow diagram (p<0.001). Librarian involvement was associated with using reporting tools (p<0.001), increased number of databases queried (p<0.001), describing date of database search (p=0.002), mentioning search peer reviewer (p=0.02), and reproducibility of search strategies (p<0.001). For search strategy quality, librarian involvement was associated with greater use of “Boolean & proximity operators” (p=0.004), “subject headings” (p<0.001), “text word searching” (p<0.001), and “spelling/syntax/line numbers” (p<0.001). Studies with librarian involvement were associated with publication in journals with higher impact factors for 2015 (p=0.003) and 2021 (p<0.001).

**Conclusion:**

Librarian involvement was associated with improved reporting quality and search strategy quality. Our study supports the inclusion of librarians in review teams, and journal editing and peer reviewing teams.

## INTRODUCTION

Systematic reviews, including meta-analyses, have become a hallmark of holistically unifying research. For health disciplines, these studies were first established in the early 1990s by the founding of the Cochrane Collaboration [1]. Despite the increase in quantity over the past few years, systematic review quality and adherence to reporting standards have remained highly variable [2-4].

As the quantity of systematic review publications increased and formalized guidelines were established, the services of medical librarians (also known as health information professionals or medical information specialists) have evolved to encompass and facilitate these studies [5]. Oftentimes, the medical librarian's role in research is assumed to be mainly focused on knowledge organization and access. However, librarians have expertise in conducting literature searches, managing citations, creating data extraction and quality assessment forms, peer-reviewing searches, writing or editing portions of manuscripts, performing statistical analyses, or acting as methodology consultants for research teams [6, 7]. In addition to contributing expertise, librarians spend a considerable amount of time on systematic review tasks and do not always receive recognition for their efforts [8, 9]. For example, a study found that librarians spend an average of 26.9 hours (median 18.5 hours) for a single systematic review [8].

Many organizations that guide best practices for systematic reviews recommend involving librarians in the research process. The National Academies of Sciences, Engineering, and Medicine (formally the Institute of Medicine) recommends working with a librarian or other information specialist to plan and peer review the search strategy [10]. Likewise, the Cochrane Collaboration recommends that review authors seek guidance from a medical librarian on the development and documentation of the search strategy [11]. The Medical Library Association (MLA) released a statement, which was cosigned by the Canadian Health Libraries Association/Association des bibliothèques de la santé du Canada (CHLA/ABSC), advocating for librarian co-authorship on evidence synthesis publications, including guidelines and systematic reviews [12]. A strong and comprehensive systematic review search strategy can ameliorate several types of reporting biases, including publication bias, language bias, citation bias, outcome reporting bias, time-lag bias, and location bias [10, 13]. These recommendations for librarian collaboration on systematic reviews aim to increase adherence to reporting guidelines and improve systematic review search quality.

In response to these recommendations, several studies have examined the value of including librarians in the systematic review process. These studies found low rates of librarian acknowledgment or co-authorship, yet involvement of librarians yielded improved search quality, better adherence to reporting standards, and lower risk of bias [1, 4, 6, 13-15]. Several of these studies were limited to certain journals within one or a few medical specialties (e.g., dentistry, cardiology, or pediatrics), and none have examined otolaryngology [16]. Additionally, many of these studies were published before 2019, and numerous systematic reviews were conducted after this time. This study addresses the gap in published literature for otolaryngology researchers and clinicians, provides further justification for the inclusion of librarians on otolaryngology systematic review teams, and contributes evidence of quantifiable changes in search strategy quality when medical librarians are involved. Therefore, our study aims to 1) elucidate the systematic review reporting quality and literature search quality of otolaryngology literature, and 2) investigate the effect of librarian involvement on search quality, search reproducibility, and systematic review reporting in otolaryngology systematic reviews and meta-analyses.

## METHODS

### Study Design and Participants

For this retrospective cross-sectional study, otolaryngology journals were selected using Journal Citation Reports™ [17]. From the journals in the “OTORHINOLARYNGOLOGY – SCIENCE” category, three researchers (MS, TG, TM) independently reviewed and selected journals based on pre-defined eligibility criteria. Inclusion criteria consisted of English language, clinically focused, otolaryngology specific, and indexed in MEDLINE. The librarians (EB, RW) identified the journals that were indexed in MEDLINE, as these journals passed the rigorous, multi-step, quality control process required by the National Library of Medicine [18]. Journals were excluded if they were non-English language, non-clinically focused, non-otolaryngology specific, and not indexed in MEDLINE. Non-English language articles were excluded due to the lack of funding for translation services or reliable translation software.

PubMed was queried to identify systematic reviews and meta-analyses in included otolaryngology journals. To identify trends over time, studies from 2010, 2015, and 2021 were included. Due to the number of articles retrieved, each year was limited to a period of six months, beginning January 1 and ending June 30. Publication dates were determined by using the “Custom Range Publication Date” filter, equivalent to using the [dp] or [pdat] field tags, in PubMed. The full search strategy is shown in Appendix A.

Retrieved articles were uploaded to Covidence systematic review software for screening [19]. Two researchers (MS, TG) independently performed title/abstract screening followed by full-text screening using pre-defined inclusion and exclusion criteria. Articles were included if the article title, abstract, or text indicated the study was a systematic review or meta-analysis; the articles were published in the selected otolaryngology journals; and the article was published between 1/1/10 - 6/30/10, 1/1/15 - 6/30/15, 1/1/21 - 6/30/21. Articles were excluded if they discussed a basic science topic, were non-English language or if the full text was irretrievable. Full-text articles were retrieved via library subscriptions, interlibrary loan, and outreach to authors.

### Data Collection

Two researchers (MS, TG) independently extracted data from the selected articles using a customized data extraction form (Appendix B). The two librarians (EB, RW) provided consensus over any disagreements in the original data extraction process. The following data elements were extracted: journal name, publication type, level of librarian involvement, reporting guideline followed, number of databases searched, dates of database searches, database limits and filters, search peer review by a second librarian, flow diagram inclusion, grey literature searched, and citation searching performed. Journal impact factors were collected for 2010, 2015, and 2021 according to Journal Citations Report™ [17]. If supplemental files containing search strategies were missing from the journal website, corresponding authors were contacted in an attempt to obtain those files.

In this study, four types of librarian involvement were identified: no acknowledgment, mentioned in text, acknowledgment, and co-authorship. “No acknowledgment” indicated that a librarian was not mentioned in the text, acknowledgments, or author byline. For “librarian mentioned in the text,” authors specified in the text of the article, normally the methods section, that a librarian assisted with search strategy development. “Librarian acknowledgment” was defined as a formal acknowledgment at the end of a manuscript. The final type, “librarian co-authorship,” means a librarian was identified in the author byline. This determination was made by examining author credentials or degrees, departmental affiliations, or by searching author names in institutional directories.

Two librarians (EB, RW) independently rated the reproducibility and quality of the search strategy for each included article. A reproducible search strategy was defined as a search strategy that was sufficiently described and could be replicated in the appropriate database with minimal effort. This would include fully described search strategies, or a combination of features of reproducible search strategies. These features included, but were not limited to, PICO tables, keywords, and Boolean operators. For articles that included at least one reproducible search strategy, six elements were rated: 1) Translation of the research question, 2) Boolean & proximity operators, 3) Subject headings, 4) Text word searching, 5) Spelling, syntax, and line numbers, 6) Limits and filters. These six elements were based on The Peer Review of Electronic Search Strategies (PRESS) checklist [20]. PRESS is a validated structured tool for the peer review of electronic literature search strategies. Each of the six elements is rated “no revisions,” “revisions suggested,” or “revisions required.” For our study, this scale was adapted to a Likert scale, ranging from 1 (low quality) to 3 (high quality) [21]. See Appendix C for the search quality form. Articles where the search was conducted by an author of this study were blinded and sent to two additional librarians (CA, IL) for quality assessment.

### Data Analysis

All data were analyzed using SPSS v27.0.1 (IBM Corporation, Armonk, NY). For Likert scale ratings on search strategy quality, interrater agreement was assessed via Cohen's Kappa statistic. Level of interrater agreement was classified according to Landis and Koch's criteria [22]. The Likert scale scores provided by each librarian were averaged for analyses.

All data were assessed for normality via Shapiro-Wilk Tests. Categorical data were presented as counts (% whole) and compared with Chi-Squared tests. For analyses of two groups vs. two groups, and one grouping had fewer than 10 counts, Fisher's Exact test was used instead of Chi-Squared. Continuous variables were presented as median (25-75% interquartile range) and compared via Mann Whitney U Tests for two groups, and Kruskal-Wallis Tests for three or more groups. Because of the low number of studies from 2010, these studies were not separately analyzed in the comparison of search strategy quality.

## RESULTS

**Figure 1:**
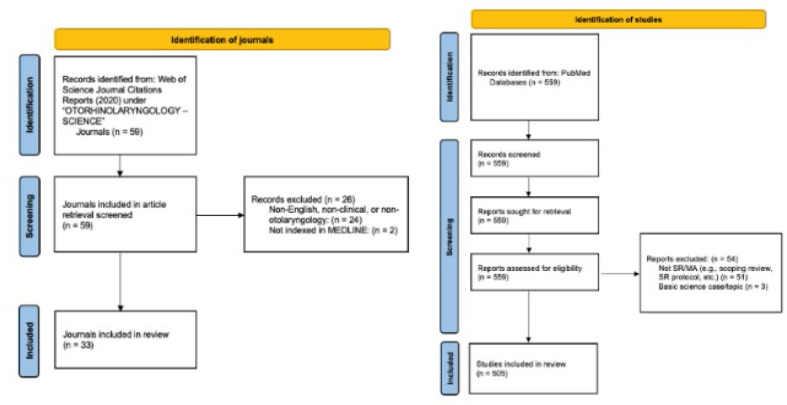
Flow diagram of journal and article inclusion.

The flow diagrams for journal and article inclusion are shown in Figure 1. Of 59 journals, 33 were included for article retrieval from PubMed. Of 559 articles retrieved, 505 were included for data extraction and analysis. All data collected did not exhibit normality.

### Temporal Changes in Reporting of Systematic Reviews and Meta-Analyses

Table 1 compares the reporting quality of systematic reviews and meta-analyses by year. Significantly more librarians were co-authors in 2021 (n=34, 9.8%) compared to 2015 (n=2, 1.5%) and 2010 (n=0, 0.0%) (p=0.04). Conversely, significantly fewer studies were unclear or did not mention librarian involvement in 2021 (n=276, 79.3%) compared to 2015 (n=121, 89.6%) and 2010 (n=20, 90.9%) (p=0.04).

**Table 1:** Comparing Year of Publication for Reporting Quality of Systematic Reviews and Meta-Analyses

	2010 (n=22)	2015 (n=135)	2021 (n=348)	Comparing All Years	Comparing 2015 to 2021
Study Type (n (%))					
Systematic Review	16 (72.7)	79 (58.5)	165 (47.4)	p = 0.005	p = 0.007
Meta-Analysis	3 (13.6)	23 (17.0)	45 (12.9)		
Systematic Review + Meta-Analysis	3 (13.6)	33 (24.4)	138 (39.7)		
Librarian Involvement (n (%))					
Co-author	0 (0.0)	2 (1.5)	34 (9.8)	p = 0.04	p = 0.01
Acknowledgement	1 (4.5)	9 (6.7)	27 (7.8)		
Mentioned in Text	1 (4.5)	3 (2.2)	11 (3-2)		
Unclear or Not Mentioned	20 (90.9)	121 (89.6)	276 (79.3)		
Systematic Review / Meta-Analysis Reporting Tool Used (n (%))					
Cochrane Handbook of Systematic Reviews of Interventions	1 (4-5)	2 (1.5)	2 (0.6)	*p*< 0.001	*p*< 0.001
Centre for Reviews and Dissemination	0 (0.0)	1 (0.7)	0 (0.0)		
MOOSE	0 (0.0)	0 (0.0)	6 (1.7)		
PRISMA	0 (0.0)	35 (25.9)	274 (78.7)		
MOOSE + PRISMA	0 (0.0)	0 (0.0)	5 (1.4)		
QUOROM	0 (0.0)	1 (0.7)	0 (0.0)		
Unclear or Unmentioned	21 (95.5)	96 (71.1)	61 (17.5)		
Number of Databases Queried					
Unclear or Not Listed	1 (4.5)	3 (2.2)	1 (0.3)	*p*< 0.001	*p*< 0.001
Mean (SD)	4.0 (4.5)	3.3 (3.2)	3.6 (1.5)		
Median (25–75 IQR)	3.0 (2.0–4.0)	3.0 (2.0–4.0)	3.0 (3.0–4.0)		
Range	1–20	1–30	1–12		
Date of Database Search Described (n (%))					
No Date Listed	7 (31.8)	31 (23.0)	48 (13.8)	*p*< 0.001	*p*< 0.001
Month, Year Listed	12 (54.5)	66 (48.9)	133 (38.2)		
Day, Month, Year Listed	3 (13.6)	38 (28.1)	167 (48.0)		
Limits/Filters Described (n (%))					
Described for at least one database	17 (77.3)	66 (48.9)	161 (46.3)	p = 0.03	p = 0.4
Unclear or Not Mentioned	1 (4.5)	7 (5.2)	10 (2.9)		
No Limits/Filters Described	4 (18.2)	62 (45.9)	177 (50.9)		
Search Strategy Peer Review Mentioned (n (%))					
Yes	0 (0.0)	0 (0.0)	4 (1-1)	p = 0.4	p = 0.58^a^
No	22 (100.0)	135 (100.0)	344 (98.9)		
Flow Chart Included (n (%))					
Yes	12 (54.5)	109 (80.7)	334 (96.0)	*p*< 0.001	*p*< 0.001
No	10 (45.5)	26 (19.3)	14 (4.0)
Grey Literature Searched (n (%))				p = 0.5	p = 0.3
Yes, Details Provided	5 (22.7)	30 (22.2)	76 (21.8)		
Yes, Details Not Provided	2 (9.1)	15 (11.1)	59 (17.0)		
No or Not Mentioned	15 (68.2)	90 (66.7)	213 (61.2)	p = 0.5 p = 0.1	p = 0.3 p = 0.3
Citation Searching Performed (n (%))					
Yes, Details Provided	7 (31.8)	58 (43.0)	175 (50.3)		
Yes, Details Not Provided	6 (27.3)	18 (13.3)	42 (12.1)		
No or Not Mentioned	9 (40.9)	59 (43.7)	131 (37.6)	p = 0.1 *p*< 0.001	p = 0.3 *p*< 0.001
Reproducibility of Search Strategy (n (%))					
No Reproducible Search Strategy Provided	15 (68.2)	78 (57.8)	128 (36.8)		
Reproducible Search Strategy for One Database	5 (22.7)	22 (16.3)	56 (16.1)		
Reproducible Search Strategies for More than One Database	2 (9.1)	35 (25.9)	164 (47.1)	*p*< 0.001	*p*< 0.001

Abbreviation: MOOSE, Meta-analyses of Observational Studies in Epidemiology; PRISMA, Preferred Reporting Items for Systematic Reviews and Meta-Analyses; QUOROM, Quality of Reporting of Meta-Analyses.

a Statistical Test modified to Fisher's Exact test (2-sided

Systematic review and meta-analysis reporting quality improved in 2021 compared to prior years in using a reporting tool (p < 0.001), number of databases queried (p < 0.001), describing the date of database searches (p < 0.001), and including a flow diagram (p < 0.001). No significant difference was seen for mentioning a peer reviewer for search strategies, searching grey literature, performing citation searching, and providing reproducible search strategies. The Preferred Reporting Items for Systematic Reviews and Meta-Analyses (PRISMA) reporting checklist [23, 24] was the most frequently used tool.

### Librarian Involvement and Study Reporting Quality

Table 2 compares the reporting of systematic reviews and meta-analyses with vs. without librarian involvement. There were statistically significant differences with regards to using a reporting tool (p < 0.001), number of databases queried (p < 0.001), describing the date of database search (p = 0.002), mentioning of a search strategy peer reviewer (p = 0.02), and reproducibility of search strategies (p < 0.001). No significant difference was seen for querying at least three databases, describing limits/filters, including a flow diagram, searching grey literature, and performing citation searching. When comparing librarians as co-authors vs. librarians involved without co-authorship, the only statistically significant difference seen was that studies involving librarian coauthors more frequently reported grey literature searching with details provided.

**Table 2:** Comparing Librarian Involvement for Reporting Quality of Systematic Reviews and Meta-Analyses

	No Librarian Involvement	Librarian Involvement^a^	Statistical Test	Librarian Co-Author (n=36)	Librarian Involved but not Co-Author^b^ (n=52)	Statistical Test
Study Type (n (%))						
Systematic Review	211 (50.6)	49 (55.7)	p = 0.02	19 (52.8)	30 (57.7)	p = 0.9
Meta-Analysis	67 (16.1)	4 (4.5)		2 (5.6)	2 (3.8)	
Systematic Review + Meta-Analysis	139 (33.3)	35 (39.8)		15 (41.7)	20 (38.5)	
Reporting Tool Used (n (%))						
No	162 (38.8)	16 (18.2)	p< 0.001	5 (13.9)	11 (21.2)	p = 0.4
Yes	255 (61.2)	72 (81.8)		31 (86.1)	41 (78.8)	
Number of Databases Queried (median [25–75% IQR])						
	3.0 (2.0–4.0)	4.0 (3.0–5.0)	p< 0.001	4.0 (3.0–5.0)	3.5 (3.0–4.75)	p = 0.2
At Least 3 Databases Queried (n (%))						
No	116 (27.8)	21 (23.9)	p = 0.5	3 (8-3)	7 (13.5)	p = 0.5
Yes	301 (72.2)	67 (76.1)		33 (91.7)	45 (86.5)	
Date of Database Search Described (n (%))						
No Date Listed	77 (18.5)	9 (10.2)	p = 0.002	2 (5.6)	7 (13.5)	p = 0.4
Month, Year Listed	183 (43.9)	28 (31.8)		11 (30.6)	17 (32.7)	
Day, Month, Year Listed	157 (37.6)	51 (58.0)		23 (63.9)	28 (53.8)	
Limits/Filters Described (n (%))						
For at least one database	203 (48.7)	41 (46.6)	p = 0.7	19 (52.8)	22 (42.3)	p = 0.6
Unclear or Not Mentioned	16 (3.8)	2 (2.3)		1 (2.8)	1 (1-9)	
None Described	198 (47.5)	45 (51.1)		16 (44.4)	29 (55.8)	
Search Strategy Peer Review Mentioned (n (%))						
No	416 (99.8)	85 (96.6)	p = 0.02^c^	35 (97.2)	50 (96.2)	p = 1.0
Yes	1 (0.2)	3 (3.4)		1 (2.8)	2 (3.8)	
Flow Chart Included (n (%))						
No	45 (10.8)	5 (5.7)	p = 0.2	1 (2.8)	4 (7.7)	p = 0.6
Yes	372 (89.2)	83 (94.3)		35 (97.2)	48 (92.3)	
Grey Literature Searched (n (%))						
Yes, Details Provided	84 (20.1)	27 (30.7)	p = 0.1	15 (41.7)	12 (23.1)	p = 0.03
Yes, Details Not Provided	64 (15.3)	12 (13.6)		7 (19.4)	5 (9.6)	
No or Not Mentioned	269 (64.5)	49 (55.7)		14 (38.9)	35 (67.3)	
Citation Searching Performed (n (%))						
Yes, Details Provided	197 (47.2)	43 (48.9)	p = 0.4	16 (44.4)	27 (51.9)	p = 0.2
Yes, Details Not Provided	51 (12.2)	15 (17.0)		4 (11.1)	11 (21.2)	
No or Not Mentioned	169 (40.5)	30 (34.1)		16 (44.4)	14 (26.9)	
Reproducibility of Search Strategy (n (%))						
None	199 (47.7)	22 (25.0)	p< 0.001	8 (22.2)	14 (26.9)	p = 0.9
Provided for One Database	67 (16.1)	16 (18.2)		7 (19.4)	9 (17.3)	
Provided for > 1 Database	151 (36.2)	50 (56.8)		21 (58.3)	29 (55.8)	

Data represented with n (%) unless otherwise specified. Chi-Squared Test was used for categorical variables

a Librarian co-author, mentioned-in text, formal acknowledgment.

b Librarian mentioned-in text, formal acknowledgment.

c Statistical test modified to Fisher's Exact test (2-sided).

### Librarian Involvement and Search Strategy Quality

Appendix D shows the descriptive statistics and interrater agreement for Likert scale ratings of search strategy quality. The greatest agreement was in “subject headings” (Kappa value 0.90 [0.89–0.94]), and the weakest was seen in “spelling/syntax/line numbers” (Kappa value 0.47 [0.38–0.56]).

Table 3 shows differences in search strategy quality grouped by levels of librarian involvement. Librarian involvement was associated with significant improvements in “Boolean & proximity operators” (p=0.004), “subject headings” (p < 0.001), “text word searching” (p < 0.001), and “spelling/syntax/line numbers” (p < 0.001). When comparing librarians as co-authors to librarian involvement without co-authorship, there were no statistically significant differences in search quality. However, trends toward significance were seen, such as in “text word searching” (p=0.06).

**Table 3:** Comparing Librarian Involvement for Search Strategy Quality

	Librarian Not Involved	Librarian Involved^a^	Mann-Whitney U Test	Librarian Co-Author	Librarian Involved but not Co-Author^b^	Mann-Whitney U Test
Studies Across All Years (Median (IQR))						
Number of Studies^c^	n = 218	n = 66	NA	n = 28	n = 38	NA
Translation of Research Question	3.0 (2.0–3.0)	3.0 (2.5–3.0)	p = 0.2	3.0 (2.5–3.0)	3.0 (2.5–3.0)	p = 0.4
Boolean & Proximity Operators	2.5 (1.5–3.0)	3.0 (2.5–3.0)	p = 0.004	3.0 (2.6–3.0)	3.0 (1.9–3.0)	p = 0.2
Subject Headings	1.0 (1.0–2.0)	3.0 (2.0–3.0)	p< 0.001	3.0 (2.5–3.0)	3.0 (1.0–3.0)	p = 0.1
Text Word Searching	2.0 (1.0–2.0)	3.0 (2.0–3.0)	p< 0.001	3.0 (2.0–3.0)	2.5 (2.0–3.0)	p = 0.06
Spelling/Syntax/Line Numbers	2.5 (2.0–3.0)	3.0 (2.4–3.0)	p< 0.001	3.0 (2.1–3.0)	2.8 (2.4–3.0)	p = 0.6
Limits/Filters	3.0 (2.0–3.0)	3.0 (2.0–3.0)	p = 0.6	3.0 (2.0–3.0)	3.0 (2.0–3.0)	p = 0.7
Overall Mean Score	2.2 (1.9–2.4)	2.7 (2.3–3.0)	p< 0.001	2.8 (2.4–3.0)	2.6 (2.2–2.9)	p = 0.2
Studies Published in 2015 (Median (IQR))						
Number of Studies	n = 50	n = 7	NA	n = 1	n = 6	NA
Translation of Research Question	2.5 (2.0–3.0)	3.0 (2.0–3.0)	p = 0.2	NA	NA	NA
Boolean & Proximity Operators	2.5 (1.0–3.0)	3.0 (1.0–3.0)	p = 0.4	NA	NA	NA
Subject Headings	1.0 (1.0–2.0)	3.0 (1.0–3.0)	p = 0.008	NA	NA	NA
Text Word Searching	1.5 (1.0–2.0)	3.0 (3.0–3.0)	p = 0.002	NA	NA	NA
Spelling/Syntax/Line Numbers	2.5 (2.0–3.0)	3.0 (1.5–3.0)	p = 0.3	NA	NA	NA
Limits/Filters	2.5 (1.5–3.0)	3.0 (2.5–3.0)	p = 0.3	NA	NA	NA
Overall Mean Score	2.0 (1.8–2.3)	2.9 (2.2–3.0)	p = 0.01	NA	NA	NA
Studies Published in 2021 (Median (IQR))						
Number of Studies	n= 161	n = 59	NA	n = 27	n = 32	NA
Translation of Research Question	3.0 (2.5–3.0)	3.0 (2.5–3.0)	p = 0.9	3.0 (2.5–3.0)	3.0 (2.5–3.0)	p = 0.4
Boolean & Proximity Operators	3.0 (2.0–3.0)	3.0 (2.5–3.0)	p = 0.01	3.0 (2.5–3.0)	3.0 (2.0–3.0)	p = 0.2
Subject Headings	1.0 (1.0–2.0)	3.0 (2.0–3.0)	*p*<0.001	3.0 (2.5–3.0)	3.0 (1.1–3.0)	p = 0.1
Text Word Searching	2.0 (1.0–2.0)	2.5 (2.0–3.0)	*p*<0.001	3.0 (2.0–3.0)	2 (2.0–3.0)	p = 0.03
Spelling/Syntax/Line Numbers	2.5 (2.0–3.0)	3.0 (2.5–3.0)	p = 0.002	3.0 (2.0–3.0)	2.5 (2.5–3.0)	p = 0.7
Limits/Filters	3.0 (2.0–3.0)	3.0 (2.0–3.0)	p = 0.7	3.0 (2.0–3.0)	3.0 (2.0–3.0)	p = 0.7
Overall Mean Score	2.3 (2.0–2.5)	2.7 (2.3–3.0)	*p*<0.001	2.8 (2.4–3.0)	2.6 (2.1–3.0)	p = 0.2

Abbreviation: NA, Not analyzed due to insufficient data. Studies published in 2010 were not analyzed separately due to an insufficient amount of data.

a Librarian co-author, mentioned-in text, formal acknowledgment.

b Librarian mentioned-in text, formal acknowledgment.

c Number of studies decreased from previous analyses because not all included studies provided a reproducible search strategy.

**Figure 2:**
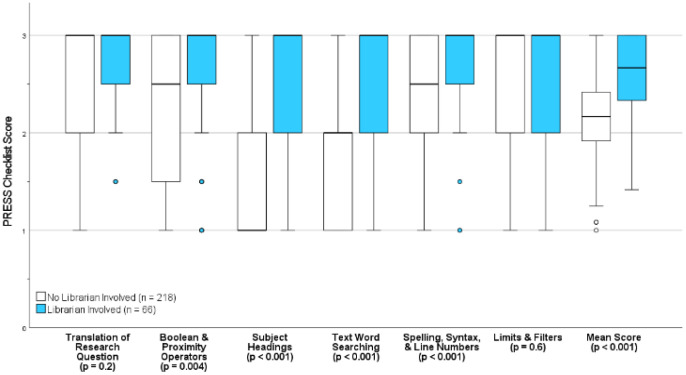
Box Plots of Median Quality of Search Strategy.

Analyses examining studies published in 2021 found improvements in search quality that mirrored analyses examining all years aggregately. These improvements again included “Boolean & proximity operators” (p=0.01), “subject headings” (p < 0.001), “text word searching” (p < 0.001), and “spelling/syntax/line numbers” (p = 0.002). When comparing librarians as co-authors to librarian involvement without co-authorship, statistically significant difference was seen with librarian co-authors for “text word searching” (p = 0.03). Figure 2 shows box plots illustrating the quality of search strategies comparing any librarian involvement (regardless of co-authorship) versus no librarian involvement.

Librarian Involvement and Publication Metrics

Appendix E shows differences in Journal Impact Factor when comparing different levels of librarian involvement. Journal impact factors were higher for articles with librarian involvement in 2015 (p = 0.003) and 2021 (p < 0.001). Impact factors were not higher with for articles published with librarian co-authors as compared to librarian involvement without co-authorship (2010 p = 0.03, 2015 p = 0.3, 2021 p = 0.9).

## DISCUSSION

Our study provided the first investigation of librarian collaboration and temporal changes in otolaryngology systematic reviews and meta-analyses. In comparing studies published in 2010, 2015, and 2021, there were significant improvements in adherence to reporting standards. However, several deficits in consistent reporting were still noted in 2021. There was significantly more collaboration with librarians, which could in part account for some improvements noted between years. Librarian involvement was associated with several statistically significant improvements in reporting quality of systematic reviews, as well as search strategy quality. Studies involving librarians were generally published in journals with higher impact factors. There was no statistically significant difference in journal impact factors between studies published with librarian co-authors compared to studies with librarians involved but not co-authors. However, some p-values approached but did not reach 0.05, which suggested that additional data and greater power could have led to statistically significant differences in journal impact factors between studies with librarians involved as co-authors and those with librarians in other roles.

### Reporting Quality of Systematic Reviews in Otolaryngology

Reporting tools were designed to facilitate transparent, complete, and accurate reporting of systematic reviews and meta-analyses. Table 1 shows that the use of a reporting tool increased from 28.9% in 2015 to 82.5% in 2021. Despite this increase, our study found significant discrepancies in proper adherence. Based on Table 1, adherence to the PRISMA checklist and PRISMA for Searching (PRISMA-S) extension was inadequate in 2021, as 36.8% did not provide a reproducible search strategy, and 52.0% did not provide the exact date that database searching was conducted. Some areas of reporting were adequately addressed by 2021, including describing limits and filters (97.1%) and using a flow diagram (96.0%). However, 61.2% did not report whether they searched grey literature, 37.6% did not report whether they performed citation searching, and 20.4% did not query at least 3 databases.

Another consideration when comparing the reporting quality in systematic reviews is the major revision that was made to PRISMA in 2020 [23]. Articles published in 2015 would have utilized the PRISMA 2009 reporting guidelines, while articles published in 2021 may have used either the original 2009 guidelines, or the revisions released in 2020. The major revisions to PRISMA in 2020 should not have impacted whether a systematic review reported the utilization of a reporting tool.

The reporting recommendations by PRISMA and the Cochrane Handbook for Systematic Reviews of Interventions provide structured guidance on the methodology and reporting of comprehensive literature searches [11, 23-25]. As such, lack of adherence to reporting tools in systematic reviews and meta-analyses may result in the omission of potentially relevant articles due to a lower quality search strategy. Regardless of librarian involvement, the conduct and reporting standards of systematic reviews in otolaryngology could benefit from stricter publication criteria and pre-publication screening.

### Librarian Collaboration and Reporting Quality

Concordant with the literature for other medical specialties, our study found that otolaryngology systematic reviews with better reporting quality were associated with librarian involvement. Several studies have demonstrated the benefits of including librarians in systematic review teams. Specifically, librarian collaboration was associated with a higher likelihood of: searching at least three databases, providing at least one reproducible search strategy, including more search terms in search strategies, better reporting scores in methodology sections, better search quality, presenting flow diagrams, and searching grey literature [1, 4, 6, 13, 14]. However, our study found that librarians were only involved in 20.7% of otolaryngology systematic reviews published in 2021, and less than half of these (9.8%) included librarian co-authorship. This low level of involvement could be attributed to lack of recognition towards librarian contributions, sometimes referred to as invisible labor [9].

One potential influence on systematic review search quality were the revisions to PRISMA in 2020, specifically whether a reproducible search strategy was provided for more than one database [23]. This was because the original 2009 PRISMA guidelines only required reviews to “present the full electronic search strategy for at least one database” while the 2020 update required reviews to “present the full search strategies for all databases, registers, and websites” [23, 24].

Considering that librarian services are not uniformly available for researchers, mandating the inclusion of librarians in systematic reviews would likely widen disparities in academic publishing. However, librarians have the expertise to peer review search methodology, and their inclusion on peer review teams would help to ensure adherence to reporting tools and reduce the risk of bias. Otolaryngology journals could consider incorporating librarians as reviewers of systematic reviews to further ensure scientific reporting integrity. This suggestion is further supported by a study indicating a high level of interest by medical librarians to serve as peer reviewers for academic journals which led to the creation of a Librarian Peer Review Database [26, 27].

### Librarian Collaboration and Search Strategy Quality

To quantify search strategy quality, our study adapted the PRESS checklist. Current methods of grading search strategy quality are affected by a grader's expertise and skill. Furthermore, a level of subjectivity is introduced by using the PRESS checklist [20]. Additional studies are needed to better develop objective and quantitative search strategy grading tools. Our study demonstrated moderate to substantial interrater agreement which was determined to be sufficient for further analyses.

Concordant with the literature, our study found that librarian collaboration was associated with improved search strategy quality [1, 13]. With librarian involvement the median score almost always was “3,” which indicated “no revisions necessary.” In contrast, most studies without librarian involvement required significant revisions for “subject headings,” and revisions suggested for “text word searching” and “spelling/syntax/line numbers.” Furthermore, conclusions were consistent when examining only studies published in 2021. Altogether, our study again supports the incorporation of librarians on systematic review and journal editing teams.

We initially hoped to examine the value of peer reviewing search strategies and its association with search strategy quality. However, analysis was not appropriate considering the inadequate number of articles indicating search strategy peer reviewing. Instead, we used librarian co-authorship as a surrogate indicator for greater involvement and investment in search strategy development. Previous literature has noted that librarians as co-authors are associated with improved search strategy quality compared to librarians only mentioned in the text or acknowledged [13]. For our study, search strategy quality mostly did not differ between studies involving librarians as co-authors vs librarians without co-authorship. However, grading scores were generally higher for studies involving librarian co-authors, and a statistically significant increase was seen for studies published in 2021 for “text word searching.” These trends suggest that additional data and greater power could lead to more statistically significant differences.

### Librarian Collaboration and Publication Metrics

Our study found that systematic reviews published in 2021 with librarian involvement were associated with publication in otolaryngology journals with higher impact factors. There was no statistically significant association between librarian involvement and high impact factor journals for the systematic reviews published in 2015, but this may be because the study was underpowered. It must be noted that impact factors are not synonymous with the prestige or reputation of a journal. Additionally, access to librarian services varies between researchers, and thus the observed differences may be due to other factors related to resource availability. As our study found that librarian involvement in systematic reviews was associated with higher search quality, this finding may indicate that librarian collaboration may be associated with publication acceptance in a higher impact journal but further research to confirm this is required. Nonetheless, our study supports the collaboration with librarians for systematic reviews and meta-analyses when possible.

### Study Limitations

Because of our strict inclusion and exclusion criteria, our study findings are not generalizable to other databases, languages, or non-clinically focused articles. Non-English language articles were not included in our study and may have excluded additional systematic reviews involving librarian involvement. Additionally, a very specific strategy was used in PubMed to identify systematic reviews and meta-analyses. This method may have excluded articles that were not yet indexed in MeSH or did not self-identify in their title.

This study was dependent on the information published in the articles we reviewed. If a librarian created a search strategy but that information was not stated in the article, the article would have been miscategorized. Librarians are not always credited as authors even if their contributions are in accordance with the International Health Library Associations to International Committee of Medical Journal Editors (ICMJE) authorship criteria [28]. These types of omissions could have led to the underestimating the level of librarian collaboration and the influence of librarian contributions on otolaryngology systematic reviews.

It is important to acknowledge that the PRISMA reporting guidelines originally published in 2009 were revised and updated in 2020 [23, 24]. Updates to the checklist included changes to incorporate more inclusive language, clarifying wording, and requiring a full search strategy for all databases [9, 23]. These changes may have impacted the number of reproducible searches in 2021 to be higher than in previous years. Additionally, the PRESS checklist was published in 2016 and may have indirectly led to an improvement in search quality as institutions may have utilized an internal librarian peer review process that was not mentioned in the text [20].

Many of our statistical comparisons did not show statistical significance. It is important to note that our study did not conduct a power analysis, and thus the minimum number of studies that had to be examined to achieve statistical significance was not pre-determined. As such, a lack of statistical significance may not mean that the relationship does not exist, but rather that another study of greater power may be needed.

## Conclusions

Our study provided the first investigation of temporal changes and librarian activity in otolaryngology systematic reviews and meta-analyses. Despite the frequent indication of using reporting tools, several deficits in adequate reporting were still noted in 2021. Librarian collaboration remains sparse in otolaryngology systematic reviews and meta-analyses. However, librarian involvement was associated with improved reporting quality and search strategy quality. Studies involving librarians were also published in journals with higher impact factors.

As the landscape begins to shift towards embracing librarian involvement on systematic reviews through the support of leading systematic review entities (e.g., Cochrane and Johanna Briggs Institute) and national organizations (e.g., MLA and CHLA/ABSC), we are hopeful that librarians will be invited to systematic review teams and as a part of the journal peer review process. The publication and growing awareness of additional structured guidance on systematic reviews, such as the PRISMA-S extension and the validated PRESS checklist, provides an opportunity to further increase search quality and reproducibility. Future research should include studies more directly examining the quality of recent systematic reviews with librarian co-authors compared to librarian involvement without co-authorship. Additionally, similar studies of systematic review quality and librarian involvement are needed in other disciplines.

## Data Availability

Raw data for quality assessment and data extraction are stored in Open Science Framework (https://osf.io/pdz3m/).

## SUPPLEMENTAL FILES

**Appendix A:** Full Search Strategy

**Appendix B:** Data Extraction form from Covidence

**Appendix C:** Quality Assessment form

**Appendix D:** Table: Agreement Between Two Librarian Raters

**Appendix E:** Table: Comparing Librarian Involvement for Publication Metrics
