# Supplementary material for: Effect of librarian collaboration on otolaryngology systematic review and meta-analysis quality
Source: J Med Libr Assoc. 2024 Jul 29;112(3):261–74. doi: 10.5195/jmla.2024.1774 (PMC11412119; doi:10.5195/jmla.2024.1774)
Supplement: Supplementary file 1 — Appendix A: Full Search Strategy [file jmla-112-3-261-s01.docx]

**Supplemental Appendix 1 – Full Search Strategy**

**PubMed search strategy:**

(("JAMA Otolaryngol Head Neck Surg"[Journal] OR "Int Forum Allergy Rhinol"[Journal] OR "Rhinology"[Journal] OR "Ear Hear"[Journal] OR "Otolaryngol Head Neck Surg"[Journal] OR "Dysphagia"[Journal] OR "Otolaryngol Clin North Am"[Journal] OR "Laryngoscope"[Journal] OR "Trends Hear"[Journal] OR "Head Neck"[Journal] OR "Clin Otolaryngol"[Journal] OR "Eur Arch Otorhinolaryngol"[Journal] OR "Am J Rhinol Allergy"[Journal] OR "J Otolaryngol Head Neck Surg"[Journal] OR "J Vestib Res"[Journal] OR "Otol Neurotol"[Journal] OR "Acta Otorhinolaryngol Ital"[Journal] OR "Int J Audiol"[Journal] OR "Eur Ann Otorhinolaryngol Head Neck Dis"[Journal] OR "Curr Opin Otolaryngol Head Neck Surg"[Journal] OR "Auris Nasus Larynx"[Journal] OR "Audiol Neurootol"[Journal] OR "Braz J Otorhinolaryngol"[Journal] OR "Am J Otolaryngol"[Journal] OR "Ear Nose Throat J"[Journal] OR "Int J Pediatr Otorhinolaryngol"[Journal] OR "Ann Otol Rhinol Laryngol"[Journal] OR "ORL J Otorhinolaryngol Relat Spec"[Journal] OR "Acta Otolaryngol"[Journal] OR "Am J Audiol"[Journal] OR "J Laryngol Otol"[Journal] OR "J Int Adv Otol"[Journal] OR "Folia Phoniatr Logop"[Journal]) AND ("Systematic Review"[Publication Type] OR "systematic review*"[Title] OR "meta analysis"[Publication Type] OR "meta analysis"[Title])) AND (2010/1/1:2010/6/30[pdat] OR 2015/1/1:2015/6/30[pdat] OR 2021/1/1:2021/6/30[pdat])

- Filters/limits: 559 total with all date ranges
  - 1/1/10-6/30/10: 32
  - 1/1/15-6/30/15: 154
  - 1/1/21-6/30/21: 373
- Date searched: September 3, 2021
- # of records identified: 559 total
