# Supplementary material for: Effect of librarian collaboration on otolaryngology systematic review and meta-analysis quality
Source: J Med Libr Assoc. 2024 Jul 29;112(3):261–74. doi: 10.5195/jmla.2024.1774 (PMC11412119; doi:10.5195/jmla.2024.1774)
Supplement: Supplementary file 2 — Appendix B: Data Extraction form from Covidence [file jmla-112-3-261-s02.docx]

**Supplemental Appendix 2 - Data Extraction Form from Covidence**

**Journal name:**

Full journal title preferred.

**Publication type:**

Publication type as identified by authors.

1. Systematic Review
2. Meta-analysis
3. Systematic review and meta-analysis

**Level of librarian involvement:**

Choose highest level of involvement.

1. Librarian only mentioned in text (e.g., methods section)
2. Librarian acknowledgement
3. Librarian co-author
4. Unclear or not mentioned

**Reporting guideline followed:**

As indicated by authors.

1. PRISMA (Preferred Reporting Items for Systematic Reviews and Meta-Analyses)
2. MOOSE (Meta-analyses Of Observational Studies in Epidemiology)
3. PRISMA and MOOSE
4. Unclear or not mentioned
5. Other

**Number of journal article databases searched:**

Indicate number in # format. Put "unclear" if number is not specified.

**Dates of database searches:**

The date the database search was executed.

1. No date listed
2. Approximate date listed (month and year) for at least one database
3. Exact date listed (month, day, and year) for at least one database
4. Unclear or not mentioned

**Database limits/filters described:**

(e.g., PubMed filters for publication dates, language, age. Different from inclusion/exclusion criteria.)

1. No limits/filters described
2. Yes, limits/filters described (for at least one database)
3. Unclear or not mentioned

**Peer review of database search by another librarian:**

1. No search peer review mentioned, or unclear
2. Yes, search peer review mentioned (for at least one database)

**Flow chart included:**

Flow chart in either in article text or appendix.

1. No flow chart included
2. Yes, flow chart included
3. Unclear

**Grey literature searched:**

(e.g., conference proceedings, clinical trials, journal TOCs)

1. No or not mentioned
2. Yes, but details NOT provided
3. Yes and details provided
4. Unclear

**Citation searching performed:**

(hand-searching references of relevant articles as well as citing articles)

1. No or not mentioned
2. Yes, but details NOT provided
3. Yes and details provided
4. Unclear

**Times cited:**

DO NOT FILL OUT; THIS WILL BE ENTERED BY LIBRARIANS. Number of times the article is cited according to Web of Science Core Collection 2020. Indicate number in # format. Put "unclear" if number is not specified.
