# Supplementary material for: Effect of librarian collaboration on otolaryngology systematic review and meta-analysis quality
Source: J Med Libr Assoc. 2024 Jul 29;112(3):261–74. doi: 10.5195/jmla.2024.1774 (PMC11412119; doi:10.5195/jmla.2024.1774)
Supplement: Supplementary file 3 — Appendix C: Quality Assessment form [file jmla-112-3-261-s03.docx]

**Supplemental Appendix 3 – Quality Assessment Form**

| **Covidence #** Enter number with # symbol.  Response (free text): ______ |
| --- |
| **Study ID**  Author last name and year. E.g., Gagnon 2020.  Response (free text): ______ |
| **Reproducibility of the Search Strategy** (select one).  ___ No reproducible search strategy (limited keywords only)  ___ Reproducible search strategy for one database only  ___ Reproducible search strategies for more than one database |
| **Translation of Research Question**  Search concepts clearly match the research question (e.g., PICO elements). Appropriate number of records retrieved (select one).  1 2 3  Low Quality ____ ____ _____ High quality |
| **Boolean & Proximity Operators**  Boolean operators/nesting/parentheses are used correctly and appropriately. Proximity operators are used correctly and appropriately (select one).  1 2 3  Low Quality ____ ____ _____ High quality |
| **Subject Headings**  Both subject headings and keywords are used for each search concept. Subject headings are relevant and appropriate (consider: major concept, explosion). Subheadings are used correctly and appropriately (select one).  1 2 3  Low Quality ____ ____ _____ High quality |
| **Text Word Searching**  Keywords are appropriate (e.g., synonyms & antonyms, abbreviations, spelling variants). Keyword modifiers are used correctly (e.g., truncation, field tags, quotes) (select one).  1 2 3  Low Quality ____ ____ _____ High quality |
| **Spelling, Syntax, & Line Numbers**  Keywords are spelled correctly. Search syntax is correct. Are there any errors in system syntax; for example, the use of a truncations symbol from a different search interface? Line numbers are correct and search concepts are combined correctly (select one).  1 2 3  Low Quality ____ ____ _____ High quality |
| **Limits & Filters**  Limits and filters are used correctly and appropriately (select one).  1 2 3  Low Quality ____ ____ _____ High quality |
