# Supplementary material for: Effect of librarian collaboration on otolaryngology systematic review and meta-analysis quality
Source: J Med Libr Assoc. 2024 Jul 29;112(3):261–74. doi: 10.5195/jmla.2024.1774 (PMC11412119; doi:10.5195/jmla.2024.1774)
Supplement: Supplementary file 4 — Appendix D: Table: Agreement Between Two Librarian Raters [file jmla-112-3-261-s04.docx]

**Supplemental Appendix 4 – Table: Agreement Between Two Librarian Raters**

Agreement Between Two Librarian Raters

| N = 284 | **Mean (SD)** | **Median (25-75% IQR)** | **Kappa Value [95% CI]** |
| --- | --- | --- | --- |
| **Translation of Research Question** | 2.6 (0.6) | 3.0 (2.0 – 3.0) | 0.60 [0.51 – 0.69] |
| **Boolean & Proximity Operators** | 2.4 (0.8) | 3.0 (2.0 – 3.0) | 0.78 [0.72 – 0.84] |
| **Subject Headings** | 1.7 (0.9) | 1.0 (1.0 – 3.0) | 0.90 [0.87 – 0.94] |
| **Text Word Searching** | 1.9 (0.8) | 2.0 (1.0 – 3.0) | 0.77 [0.71 – 0.83] |
| **Spelling / Syntax / Line Numbers** | 2.4 (0.7) | 2.5 (2.0 – 3.0) | 0.47 [0.38 – 0.56] |
| **Limits/Filters** | 2.5 (0.7) | 3.0 (2.0 – 3.0) | 0.67 [0.59 – 0.75] |
| **Total** | 13.5 (2.8) | 14.0 (12.0 – 16.0) | NA |

Abbreviation: NA, Not Analyzed.

Data shown from 284 articles that provided at least one reproducible search strategy. 201 articles provided search strategies for more than one database, whereas 83 articles provided a search strategy for only one database. When analyzing these groups separately, kappa values were consistent with the grouped findings.

Level of interrater agreement was classified according to Landis and Koch’s criteria as follows: slight (0.01–0.20), fair (0.21–0.40), moderate (0.41–0.60), substantial (0.61–0.80), and almost perfect (0.81–0.99).
