# Supplementary material for: Effect of librarian collaboration on otolaryngology systematic review and meta-analysis quality
Source: J Med Libr Assoc. 2024 Jul 29;112(3):261–74. doi: 10.5195/jmla.2024.1774 (PMC11412119; doi:10.5195/jmla.2024.1774)
Supplement: Supplementary file 5 — Appendix E: Table: Comparing Librarian Involvement for Publication Metrics [file jmla-112-3-261-s05.docx]

**Supplemental Appendix 5 – Table: Comparing Librarian Involvement for Publication Metrics**

Comparing Librarian Involvement for Publication Metrics

|  | **Librarian Not Involved** | **Librarian Involved^a^** | **Mann-Whitney**  **U Test** | **Librarian**  **Co-Author** | **Librarian Involved but not Co-Author^b^** | **Mann-Whitney**  **U Test** |
| --- | --- | --- | --- | --- | --- | --- |
| Studies Across All Years | | | | | | |
| Number of Studies | n = 417 | n = 88 | NA | n = 36 | n = 51 | NA |
| Impact Factor 2010 | 1.6 (1.2 – 2.1) | 1.6 (1.3 – 2.1) | p *=* 0.08 | 1.6 (1.1 – 2.1) | 2.1 (1.6 – 2.1) | p *=* 0.03 |
| Impact Factor 2015 | 2.0 (1.1 – 2.3) | 2.0 (1.8 – 2.3) | p *=* 0.003 | 2.0 (1.3 – 2.3) | 2.1 (2.0 – 2.3) | p *=* 0.3 |
| Impact Factor 2021 | 3.0 (2.5 – 3.6) | 3.0 (2.9 – 5.6) | *p <* 0.001 | 3.0 (2.9 – 5.6) | 3.2 (2.8 – 5.6) | p *=* 0.9 |
| Studies Published in 2015 | | | | | | |
| Number of Studies | n = 121 | n = 14 | NA | n = 2 | n = 12 | NA |
| Impact Factor 2010 | 1.6 (1.2 – 2.1) | 1.8 (1.1 – 2.1) | p = 1.0 | NA | NA | NA |
| Impact Factor 2015 | 2.0 (1.6 – 2.3) | 2.1 (1.5 – 2.3) | p = 0.8 | NA | NA | NA |
| Impact Factor 2021 | 3.0 (2.5 – 3.8) | 3.0 (2.6 – 5.0) | p= 0.6 | NA | NA | NA |
| Studies Published in 2021 | | | | | | |
| Number of Studies | n = 276 | n = 72 | NA | n = 34 | n = 37 | NA |
| Impact Factor 2010 | 1.6 (1.2 – 2.1) | 1.6 (1.2 – 2.1) | p = 0.046 | 1.6 (1.2 – 2.1) | 1.6 (1.5 – 2.1) | p *=* 0.04 |
| Impact Factor 2015 | 1.8 (1.1 – 2.3) | 2.0 (1.8 – 2.3) | *p* < 0.001 | 2.0 (1.5 – 2.3) | 2.1 (2.0 – 2.4) | p *=* 0.2 |
| Impact Factor 2021 | 3.0 (2.4 – 3.2) | 3.1 (2.9 – 5.6) | *p* < 0.001 | 3.0 (2.9 – 5.6) | 3.5 (2.7 – 5.6) | p = 0.9 |

Abbreviation: NA, Not analyzed due to insufficient data.

Data presented as Median [IQR]. Studies published in 2010 were not analyzed separately due to an insufficient amount of data.
^a^Librarian co-author, mentioned-in text, formal acknowledgment.
^b^Librarian mentioned-in text, formal acknowledgment.
